# Pre-hospital lowest recorded oxygen saturation independently predicts death in patients with COVID-19

**DOI:** 10.29045/14784726.2020.09.5.3.59

**Published:** 2020-12-01

**Authors:** Kate Dillon, Chris Hook, Zoe Coupland, Pascale Avery, Hazel Taylor, Andy Lockyer

**Affiliations:** University Hospitals Bristol and Weston NHS Foundation Trust; University Hospitals Bristol and Weston NHS Foundation Trust; University Hospitals Bristol and Weston NHS Foundation Trust; University Hospitals Bristol and Weston NHS Foundation Trust; University Hospitals Bristol and Weston NHS Foundation Trust; University Hospitals Bristol and Weston NHS Foundation Trust; Great Western Air Ambulance Charity

**Keywords:** coronavirus, COVID-19, NEWS2, oxygen saturation, respiratory failure, triage

## Abstract

**Background:**

The coronavirus disease 2019 (COVID-19) results in hypoxia in around a fifth of adult patients. Severe hypoxia in the absence of visible respiratory distress (‘silent hypoxia’) is increasingly recognised in these patients. There are no published data evaluating lowest recorded pre-hospital oxygen saturation or pre-hospital National Early Warning Score 2 (NEWS2) as a predictor of outcome in patients with COVID-19.

**Methods:**

In this retrospective service evaluation, we included adult inpatients with laboratory confirmed COVID-19 who were discharged from hospital or who died in hospital between 12 March and 28 April 2020 (n = 143). Pre-hospital and in-hospital data were extracted and analysed to explore risk factors associated with in-hospital mortality to inform local triage and emergency management.

**Results:**

The lowest recorded pre-hospital oxygen saturation was an independent predictor of mortality when controlling for age, gender and history of COPD. A 1% reduction in pre-hospital oxygen saturation increased the odds of death by 13% (OR 1.13, p < 0.001). Lower pre-hospital oxygen saturation predicted mortality after adjusting for the pre-hospital NEWS2 (OR for a 1% reduction in pre-hospital oxygen saturation 1.09, p = 0.02). The pre-hospital NEWS2 was higher in those who died (Median 9; IQR 7-10; n = 24) than in those who survived to discharge (Median 6; IQR 5-8; n = 63).

**Conclusion:**

This service evaluation suggests that the lowest recorded pre-hospital oxygen saturation may be an independent predictor of mortality in COVID-19 patients. Lowest pre-hospital oxygen saturation should be recorded and used in the assessment of patients with suspected COVID-19 in pre-hospital and emergency department triage settings.

## Introduction

In December 2019, the novel coronavirus SARS-CoV-2, and resulting coronavirus disease 2019 (COVID-19), emerged in mainland China. It was declared a Public Health Emergency of International Concern on 30 January 2020, and was confirmed as a pandemic by the World Health Organisation on 11 March 2020.

COVID-19 causes a wide spectrum of clinical presentations. The majority of patients develop mild symptoms, predominantly fever and cough (Zhou et al., 2020), while 20% of patients may develop severe illness (Wu & McGoogan, 2020). The hypoxia seen in COVID-19 can occur in the absence of visible respiratory distress, and is therefore termed ‘silent hypoxia’ (Ottestad & Sovik, 2020; Ottestad et al., 2020). This is driven by immunopathogenesis resulting in ARDS-like respiratory failure and microvascular injury, and can lead to multi-organ failure and death (Giwa & Desai, 2020; Magro et al., 2020). The need for hospital admission varies from 1% in 20–29 year olds to 18% in 80+ year olds, with an overall estimated case fatality ratio of 1–2% (Verity et al., 2020).

While multiple severity scoring systems exist for the critically unwell patient, few have been validated for use in COVID-19. These often require a detailed medical history, laboratory results or radiological findings, which take time and require healthcare professionals to be patient-facing (Steinberg et al., 2020). However, early recognition of COVID-19 and prediction of severe disease are vital for patients in need of time-critical interventions, as well as timely infection control and system planning (Wee et al., 2020). Since 2012, the validated National Early Warning Score (NEWS) has been widely used to standardise recognition of the deteriorating patient (RCP, 2017). It was updated to NEWS2 in 2017 with the inclusion of a dedicated score for patients with hypercapnic respiratory failure and an additional score for new confusion (RCP, 2017). A growing body of evidence increasingly supports the use of NEWS and NEWS2 in the pre-hospital setting to predict critical illness and early in-patient mortality (Abbott et al., 2018; Martin-Rodriguez et al., 2019; Williams et al., 2016). Research into the effectiveness of NEWS2 for COVID-19 is ongoing.

In response to the pandemic, significant changes were made to the admissions process at our hospital, including the development of an Incident Triage Area (ITA) to receive all patients brought by ambulance. They were streamed rapidly to COVID-19-suspected or non-COVID-19 areas by senior Emergency Department (ED) clinicians who used clinical judgement in the absence of a formal decision-making algorithm. A retrospective service evaluation was undertaken of patients admitted to hospital who tested positive for COVID-19 to inform local triage and explore risk factors associated with mortality. Further scrutiny of patients who were triaged to non-COVID-19 areas led to the identification of pre-hospital hypoxia as a ‘red flag’ in our population. To our knowledge, few papers have yet been published on the clinical parameters of patients with COVID-19 in the pre-hospital setting.

## Methods

This retrospective service evaluation included adult inpatients (≥16 years old) from an inner-city University Hospital in South West England. Of patients presenting to the ED with respiratory symptoms (either by ambulance or self-presenting), those who required hospital admission received a SARS-CoV-2 RNA PCR test. All adult patients who were diagnosed with COVID-19 by testing positive on SARS-CoV-2 RNA on PCR at any point during their admission, and had been discharged or died between 12 March 2020 and 28 April 2020, were included in this study. Data were collected broadly to inform local clinical decisions and services, with a specific interest in the effectiveness of our ITA established from 17 March 2020.

### Ethical considerations

This study was performed as part of a service evaluation. Analysis of routinely collected retrospective clinical data was performed to determine whether any parameters could be correlated with recovery of this patient cohort and be used to improve service delivery for this patient group. All data are presented as anonymised collated data. Research Ethics Committee approval was not required.

Neither patients nor the public were involved in the design, conduct, reporting or dissemination plans of our service evaluation.

### Data collection

Epidemiological, demographic, clinical, laboratory, treatment and outcome data were extracted from electronic and paper medical reports using a centralised database, REDCap. All data were checked and entered by two physicians. Follow-up was triggered by REDCap notification of incomplete outcome data and carried out continuously during the period of data collection.

### Statistical analysis

Following data extraction into Excel, demographics, clinical information and in-hospital mortality were presented. Data were analysed in Stata 14 and results were defined as statistically significant if the *p* value was <0.05. Continuous and categorical variables were presented as median (Interquartile Range (IQR)) and n (%) respectively.

Analysis of the lowest pre-hospital recorded saturation (on arrival at scene) was performed for those patients arriving at hospital by ambulance from 12 March 2020 (excluding patients who contracted COVID-19 as an inpatient and those self-presenting).

Logistic regression was used to examine the relationship between the lowest pre-hospital oxygen saturation and in-hospital mortality adjusting for age, gender and COPD. A separate logistic regression model included the pre-hospital NEWS2, but numbers were not sufficient to adjust for age, gender and COPD.

Kaplan Meier curves were produced for all patients admitted to this hospital with a positive SARS-CoV-2 RNA PCR test up to 28 April 2020, and included all patients whether or not their outcome was known. Pre-hospital oxygen saturation values were divided into four categories, and a separate curve was produced for each category. Patients without a known outcome by 28 April 2020 were censored on this day and patients who were transferred to another unit were censored on the day of transfer, giving a larger data set of n = 133 (as previous analysis had only used patients with a known outcome). The Kaplan Meier graph for time to death censored patients on the date of discharge, and the graph for the time to discharge censored patients on the date of death. Log rank tests were used to test for associations with the categorised pre-hospital oxygen saturation values.

### Minimising bias

Two physicians were used to review patient records and enter data to ensure accuracy. Patients were identified for data collection by laboratory-confirmed infection rather than clinical suspicion. To ensure inpatient COVID-19 infection could be identified and excluded, data were collected on means of arrival at hospital, location the patient was first assessed, day of COVID-19 symptoms at admission, maximum level of care received and date of outcome. When analysing results, we controlled for anticipated confounding variables through logistic regression.

## Results

### Cohort demographics and clinical information

A total of 164 patients admitted to our university teaching hospital were diagnosed with COVID-19 and tested positive for SARS-CoV-2 RNA on PCR between 12 March 2020 and 28 April 2020. An outcome of either discharged from hospital or died in-hospital was determined for 143 patients by 28 April 2020. Of these, 109 patients arrived by ambulance and had ‘lowest pre-hospital oxygen saturation’ on air recorded by paramedics on arrival at scene. ‘Pre-hospital NEWS2’ was recorded in 87 patients on arrival at scene. Table 1 shows patient demographics, and Table 2 presents the clinical information.

**Table 1.** Patient demographics.

n = 109	
Age, years	73 (58, 81)
Male gender n (%)	61 (56)
Ethnicity Black, Asian and minority ethnic n (%)	31 (28)
Co-morbidity:	
Hypertension	48 (44)
Chronic obstructive pulmonary disease/asthma	16 (15)
Stroke	10 (9)
Diabetes mellitus	37 (34)
Chronic kidney disease	24 (22)
Ischaemic heart disease	15 (14)

Continuous data presented as median (Interquartile range (IQR)) unless otherwise stated. Categorical data presented as n and %.

**Table 2.** Clinical Information.

n = 109
Mortality, n (%)	33 (30)
Lowest recorded pre-hospital oxygen saturation, %	87 (79, 91)
Time since symptom onset, days	7 (3,10)
Length of stay in hospital of discharged patients, days	8 (3, 15)
Time from admission to death, days (n = 33)	6 (3, 11)
**NEWS2**	**n = 87**
Pre-hospital NEWS2	7 (6, 9)

Continuous data presented as median (Interquartile range (IQR)) unless otherwise stated. Categorical data presented as n and %.

## Pre-hospital oxygen saturations

### Mortality

The lowest recorded pre-hospital oxygen saturation was lower in those who died (Median 79; Interquartile Range (IQR) 74–87); n = 33) than in those who were discharged (Median 88; IQR 84–93; n = 76). This difference was statistically significant (Mann-Whitney U Test, p = 0.0001).

There was no correlation between lowest recorded pre-hospital oxygen saturation and patient age (Spearman’s Correlation coefficient = –0.0868, p = 0.3692) and no difference by gender (p = 0.7739 Mann-Whitney U Test). Patients with a past medical history of COPD presented with lower oxygen saturation (Median 82; IQR 76–88; n = 16) than those without COPD (Median 87; IQR 81–92; n = 93) (p = 0.0223 Mann-Whitney U Test).

After adjusting for age, gender and a previous diagnosis of COPD, the lowest recorded pre-hospital oxygen saturations predicted death vs discharge (Odds Ratio for a 1% reduction in lowest recorded pre-hospital saturation; 1.13 (95% CI: 1.06, 1.21), p < 0.001). This indicates a 13% (95% CI 6%, 21%) increase in the odds of in-hospital death for every 1% reduction in lowest recorded pre-hospital oxygen saturation.

### Time from admission to death

**Figure 1:**
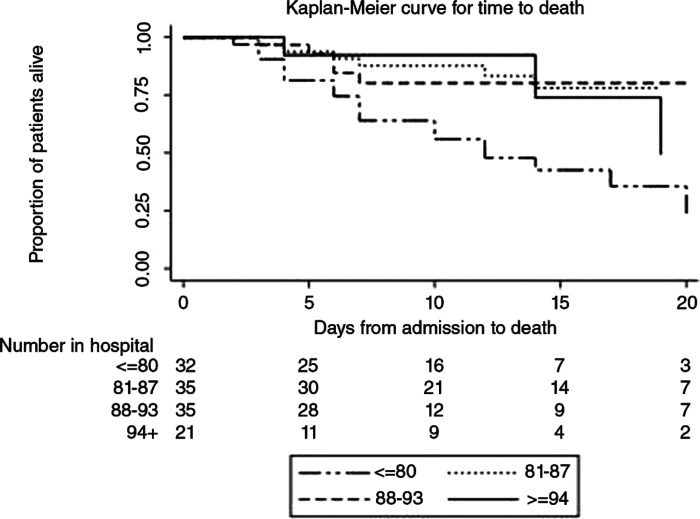
Figure 1. Time from admission to death.

Time from admission to death was significantly associated with pre-hospital oxygen saturations (Log Rank Test, 0.0015), with a trend suggesting shorter survival time for those with lower pre-hospital oxygen saturation (Log Rank Test for trend, p = 0.0046) (Figure 1).

### Intubation

Ceilings of care were identified on admission for all patients during their inpatient stay. The lowest recorded pre-hospital oxygen saturation was lower in those who were intubated (Median 78; Interquartile Range (IQR) 60–86); n = 13) than in those who were not intubated (Median 88; IQR 81–92; n = 96). This difference was statistically significant (Mann-Whitney U Test, p = 0.0045).

The number of patients intubated was too small for an adjusted analysis; unadjusted logistic regression shows a 9% increase in the odds of intubation (95% CI: 4%, 15%) for every 1% reduction in the lowest recorded pre-hospital saturation (p = 0.001).

The median time to intubation was 2 days (the day after admission). Six patients (46%) were intubated within 24 hours of admission.

### Length of admission and time from symptom onset to admission

**Figure 2:**
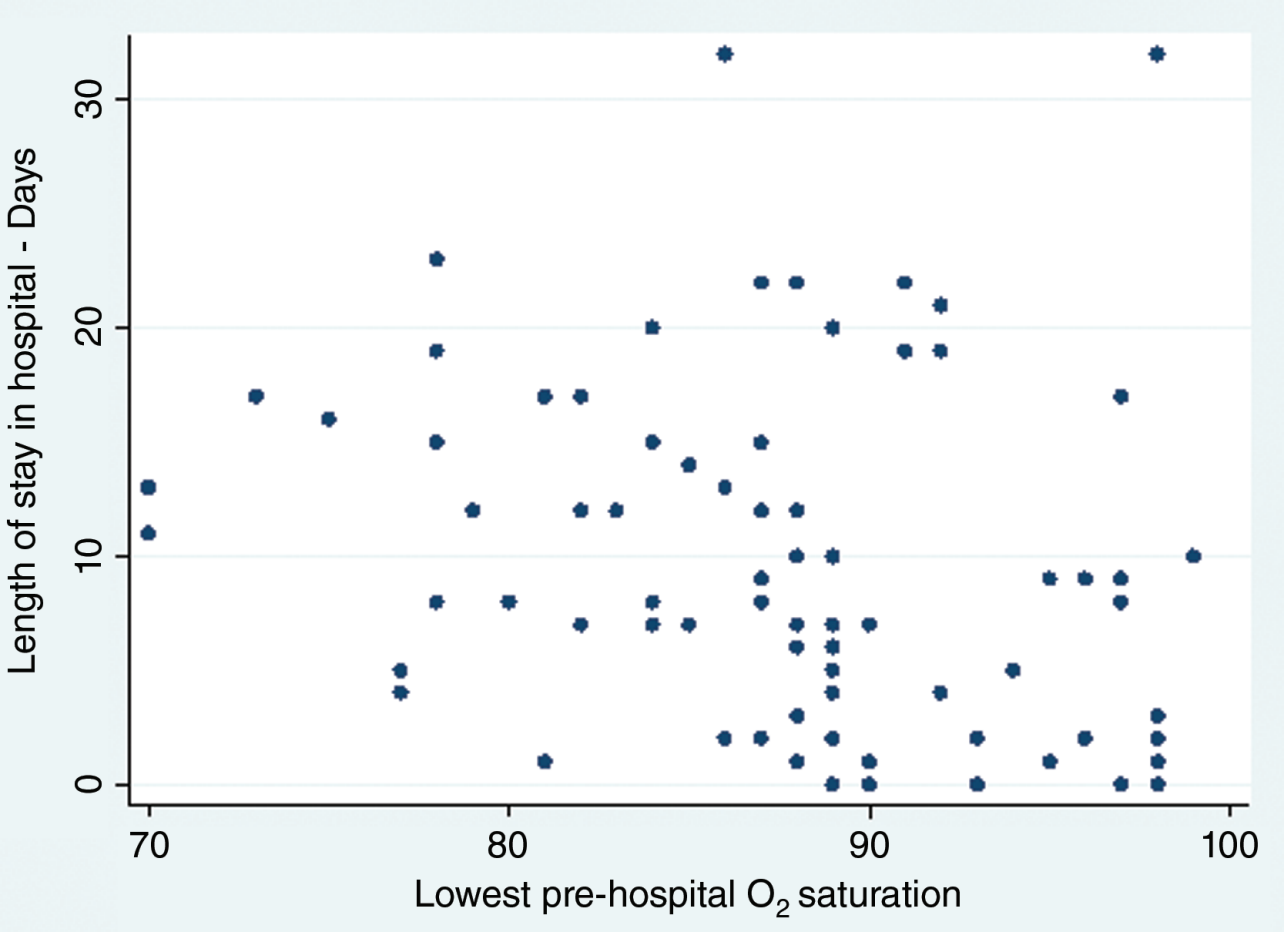
Figure 2. Pre-hospital oxygen saturation and length of hospital admission.

**Figure 3:**
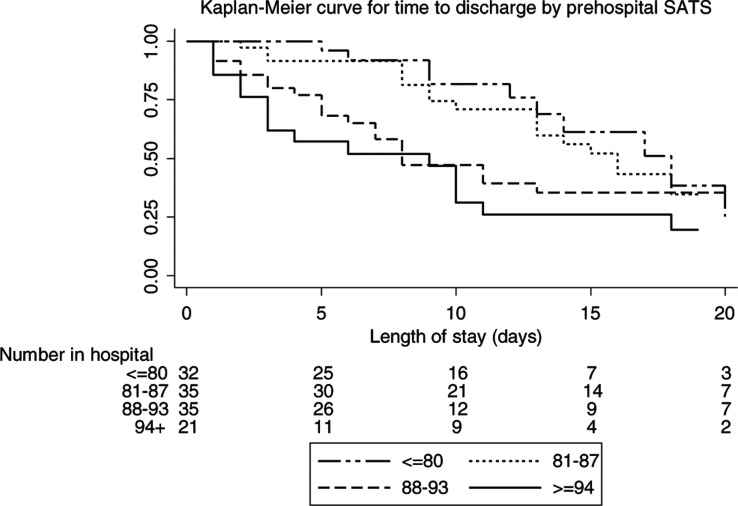
Figure 3. Time to discharge by pre-hospital oxygen saturation.

**Figure 4:**
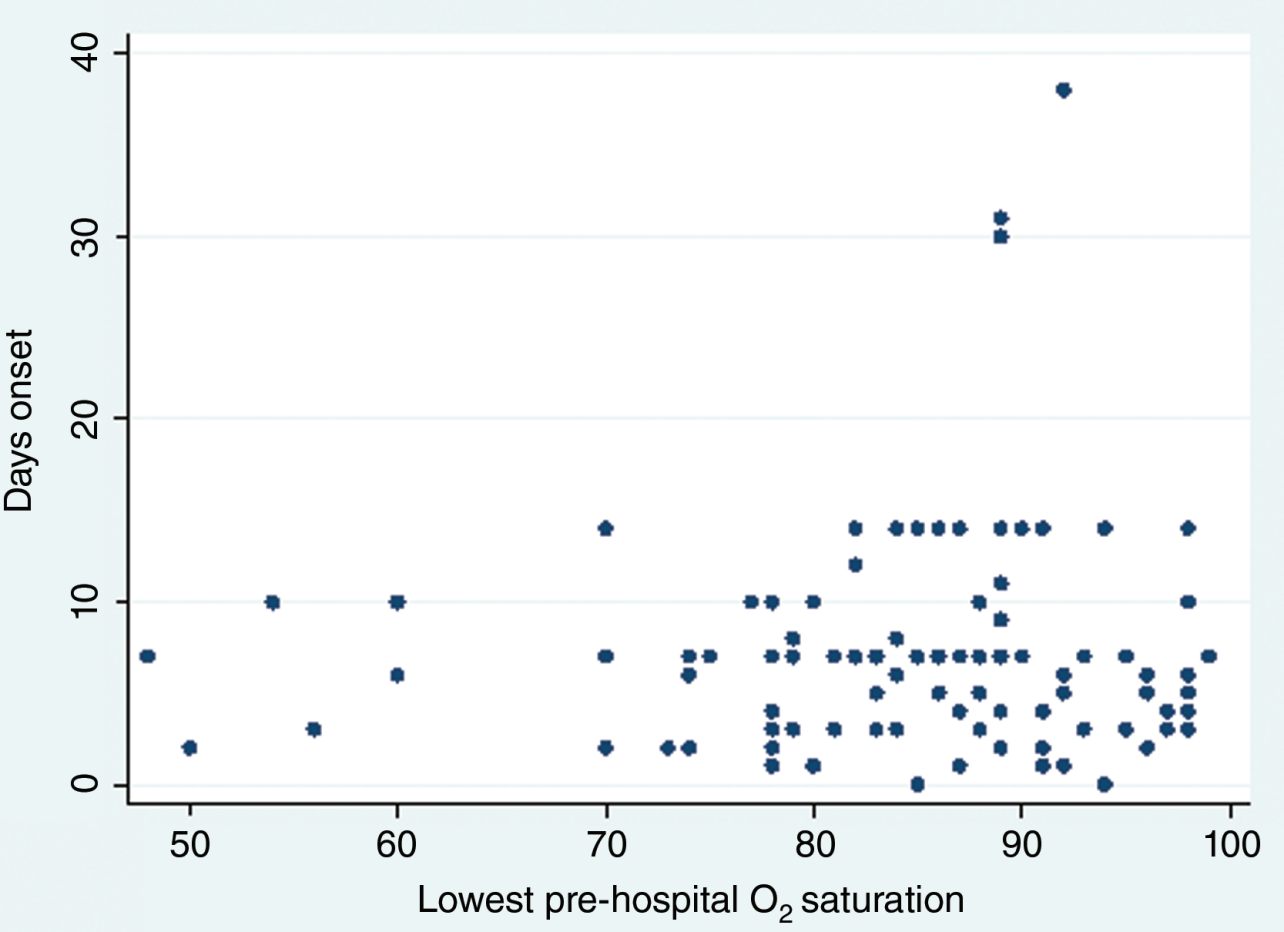
Figure 4. Pre-hospital oxygen saturation and time since symptom onset.

There is a weak negative correlation between length of stay and lowest recorded pre-hospital oxygen saturation for patients who were discharged (Spearman’s correlation = –0.3702 (n = 76), p = 0.001) (Figure 2). The Kaplan Meier curve shows patients in groups with lower pre-hospital saturation remaining in hospital longer than those in groups with higher pre-hospital saturation (Log rank test p = 0.0134; Log rank test for trend p = 0.0020) (Figure 3). There is no correlation between the time from symptom onset to admission and pre-hospital oxygen saturation (Spearman Correlation = –0.019, p = 0.8433) (Figure 4).

### Pre-hospital NEWS2

The pre-hospital NEWS2 was higher in those who died (Median 9; IQR 7–10; n = 24) than in those who survived to discharge (Median 6; IQR 5–8; n = 63). This difference was statistically significant (Mann-Whitney U Test, p = 0.0001). The pre-hospital NEWS2 was also higher in those patients who were eventually intubated (Median 9; IQR 7–10; n = 10) than those who were not intubated (Median 7; IQR 5–8; n = 77). This difference was also statistically significant (Mann-Whitney U Test, p = 0.0268).

Every patient with a pre-hospital NEWS2 score of ≤5 was eventually discharged (n = 21). There was no statistical difference in length of stay for those with a pre-hospital NEWS2 score ≤5 or ≥6.

Logistic regression shows that the lowest recorded pre-hospital oxygen saturation still predicts the odds of death after adjusting for overall pre-hospital NEWS2 (OR 1.09 for each decrease of 1% in saturation, 95% CI 1.01, 1.17, p = 0.02). This suggests that lowest pre-hospital oxygen saturation predicts mortality independently from the NEWS2.

Of interest, there was no statistical difference in respiratory rate between those who died (Median 22; IQR 19.5–28.5; n = 32) and those survived to discharge (Median 23; IQR 20–26; n = 64).

## Discussion

Internationally, NEWS2 is the most widely used bedside scoring tool available and there is increasing evidence to demonstrate that its use in the pre-hospital setting can predict critical illness and improve patient outcomes (Abbott et al., 2018; Martin-Rodriguez, Lopez-Izquierdo, et al., 2020). When introduced to pre-hospital care in the West of England, mortality reduced for patients with suspected sepsis when compared to national data (Pullyblank et al., 2020). A prospective multicentre cohort study found that use of pre-hospital NEWS2 could predict in-hospital mortality with an area under the curve (AUC) for mortality at one day of 0.862 (Martin-Rodriguez, Lopez-Izquierdo, et al., 2020), similar to a large retrospective study reporting an AUC of mortality at one day of 0.84 (Pirneskoski et al., 2019). Other studies have shown that higher pre-hospital NEWS2 are associated with critical care admission (Abbott et al., 2018; Martin-Rodriguez et al., 2019; Pirneskoski et al., 2019; Shaw et al., 2017).

Many other scoring systems have been put forward as alternatives to NEWS2. A Pre-hospital Older Adult Warning Score proposed adding in factors such as lactate and the ratio of SpO2 to FiO2 and showed promising predictive values in a small cohort of Spanish patients, although it is unlikely to be taken up across the NHS where NEWS2 is so widely utilised and point of care lactate measurements are not readily available (Martin-Rodriguez, Sanz-Garcia, et al., 2020).

NEWS2 has continued to be the predominant bedside score used during the pandemic. Recently, the Royal College of Physicians updated their NEWS2 guidance to emphasise the significance of escalating oxygen requirements in patients with COVID-19 irrespective of a change (or lack of change) in NEWS2 scores (RCP, 2020). Novel research shows that NEWS2 >7 on admission to hospital is strongly associated with critical care admission in COVID-19 (98% specificity) (Gidari et al., 2020). Our article supports the literature, finding a median pre-hospital NEWS2 of 9 among patients who died from COVID-19, while all patients with a pre-hospital NEWS2 of <5 survived to discharge.

Of note, NEWS2 may not place adequate significance on the derangement of a single parameter. Hypoxia and the use of supplemental oxygen can claim a maximum score of 5, where the highest theoretical NEWS2 score is 20. This service evaluation found that lower pre-hospital oxygen saturation was a predictor of in-hospital mortality from COVID-19, independent from NEWS2, age or gender, with a 13% increase in odds of in-patient mortality for every 1% fall in pre-hospital oxygen saturations. There is a paucity of published evidence around the significance of pre-hospital hypoxia in predicting critical illness or early mortality.

The concept of ‘silent hypoxia’ in COVID-19 is discussed in recent literature. One study found that only 17% of US adults requiring admission with COVID-19 had an abnormal respiratory rate (RR) despite 28% of patients requiring supplemental oxygen (Richardson et al., 2020). This is supported by another study which described 71% of hospitalised adults with COVID-19 in Wuhan, China as having a normal RR (Zhou et al., 2020). Another study evaluated the SpO_2_ to RR ratio in patients with acute respiratory failure and reported a significantly higher ratio in COVID-19 patients compared with non-COVID-19 patients (Jouffroy et al., 2020). This illustrates a distinct difference in COVID-19 from other forms of acute respiratory distress. This is supported by our data showing no statistical difference in RR in patients who died or survived to discharge, despite profound changes in hypoxia.

Furthermore, early recognition of silent hypoxia in suspected COVID-19 allows prevention of nosocomial spread by the appropriate use of personal protective equipment and isolation. One case report describes the late recognition of COVID-19 in an atypical presentation (abdominal pain in an elderly patient) resulting in staff infection, despite the presence of significant pre-hospital hypoxia (Ghazali et al., 2020).

The universal language of NEWS2 aims to standardise handover between clinical teams (Pullyblank et al., 2020). Research into the handover between pre-hospital and emergency clinicians has shown that human factors, such as failure to document important findings, limit its effectiveness (Wood et al., 2015). There is also evidence that clerking doctors attribute less significance to clinical observations recorded by paramedics than information recorded in hospital, and are less likely to review electronically scanned pre-hospital records than written handover reports (Knutsen & Fredriksen, 2013). It is vital that emergency and specialty physicians acknowledge abnormal pre-hospital findings, particularly where improvements in physiological derangement result from intervention. This is especially relevant to COVID-19 and the findings from this study, where the hypoxia reported in pre-hospital documentation is unlikely to be observed in the hospital setting. NHS England currently advocates the use a ‘40-step desaturation test’ to facilitate discharge for normoxic COVID-19 patients, which is thought to simulate the community setting (NHS England, 2020). A 3% reduction in oxygen saturation on exercise is considered a cause for concern (Greenhalgh et al., 2020).

## Limitations

This study has several limitations. This is a single-centre service evaluation and the findings may not be generalisable to other clinical settings or regions with different local populations. The results from this study may be influenced by confounding factors inherent in the retrospective study design, such as information bias arising from inaccurate record keeping. Missing information further limits the effectiveness of handover and may impact patient care. Only patients brought in by ambulance are included in this article, which aims to represent patients with more severe disease but does not account for socioeconomic attitudes or physical barriers to accessing healthcare.

In an attempt to obtain a true COVID-19 cohort, we included patients with laboratory PCR confirmed COVID-19 infection. However, a risk of selection bias cannot be overlooked as we did not include patients treated for clinical COVID-19 infection but with a negative PCR test. In an attempt to reduce this risk, we included those with a positive PCR result at any time during their admission (including those with a previous negative PCR result), but excluded those with hospital-acquired infection.

## Conclusion

Our retrospective service evaluation showed the lowest recorded pre-hospital oxygen saturation on arrival at scene to be an independent predictor of mortality in COVID-19 patients. Lower recordings are associated with an increased mortality and need for intubation, and a longer length of stay. This increased risk of mortality is independent of age, gender and a previous diagnosis of COPD, as well as the pre-hospital NEWS2 score. Lowest pre-hospital oxygen saturation should be recorded and used in the assessment of patients with suspected COVID-19 in pre-hospital and emergency department triage settings. Further research is required to validate these findings in prospective, multicentre studies.

## Acknowledgements

The authors acknowledge Mai Baquedano, Senior Research Associate, University of Bristol, UK; and Simon Laing, Consultant in Emergency Medicine, Bristol Royal Infirmary, University Hospitals Bristol and Weston NHS Foundation Trust, UK.

## Author contributions

Data were collected by KD, CH, ZC and PA. Material was drafted by KD, CH and ZC, and edited by PA and AL. Statistical analysis was performed by HT. The project was supervised by AL, who was also responsible for planning and conduct, and overall content. Each author has been involved in the development of the final material. AL acts as the guarantor for this article.

## Conflict of interest

None declared.

## Ethics

Not required.

## Funding

None.
